# Laboratory and Numerical Analysis of Steel Cold-Formed Sigma Beams Retrofitted by Bonded CFRP Tapes

**DOI:** 10.3390/ma13194339

**Published:** 2020-09-29

**Authors:** Ilona Szewczak, Katarzyna Rzeszut, Patryk Rozylo, Sylwester Samborski

**Affiliations:** 1 Faculty of Civil Engineering, Lublin University of Technology, 36 Nadbystrzycka Str, 20-618 Lublin, Poland; 2 Faculty of Civil and Transport Engineering, Institute of Building Engineering, Poznan University of Technology, 5 Marii Skłodowskiej-Curie Str, 60-965 Poznań, Poland; katarzyna.rzeszut@put.poznan.pl; 3 Faculty of Mechanical Engineering, Lublin University of Technology, 36 Nadbystrzycka Str, 20-618 Lublin, Poland; p.rozylo@pollub.pl (P.R.); s.samborski@pollub.pl (S.S.)

**Keywords:** thin-walled structure, FEM analysis, cold-formed steel beam, reinforcement, CFRP tape, adhesive connection

## Abstract

In this paper, the retrofitting method of thin-walled, cold-formed sigma beams using bonded carbon fibre reinforced polymer (CFRP) tapes is proposed. The effectiveness of the presented strengthening method is investigated by the means of laboratory tests and numerical analysis conducted on simply supported, single-span beams made of 200 × 70 × 2 profile by “Blachy Pruszyński” subjected to a four-point bending scheme. Special attention is paid to the evaluation of possibility to increase the load capacity with simultaneous limitation of beams displacements by appropriate location of CFRP tapes. For this purpose, three beams were reinforced with CFRP tape placed on the inner surface of the upper flange, three with CFRP tape on the inner surface of the web, three beams with reinforcement located on the inner surface of the bottom flange, and two beams were tested as reference beams without reinforcement. CFRP tape with a width of 50 mm and a thickness of 1.2 mm was used as the reinforcement and was bonded to the beams by SikaDur^®^-30 adhesive. Precise strain measurement was made using electrofusion strain gauges, and displacement measurement was performed using two Aramis coupled devices in combination with the Tritop machine. Numerical models of the considered beams were developed in the Finite Element Method (FEM) program Abaqus^®^. Experimental and numerical analysis made it possible to obtain a very high agreement of results. Based on the conducted research, it was proved how important is the impact of the applied reinforcement (CFRP tapes) in thin-walled steel structures, with respect to the classic methods of strengthening steel building structures.

## 1. Introduction

The development of thin-walled steel structures is related primarily to the technical progress in manufacturing and assembly as well as efforts to minimize material consumption. According to the definition of Vlasov, the creator of the theory of thin-walled open cross-sections, a bar can be considered thin-walled if the wall thickness is at least eight times smaller than the longest distance measured along the centre line between two extreme points located on the bar cross-section contour, and this, in turn, is at least eight times smaller than the bar length.

Compared to traditional design solutions, cold-formed elements have one of the highest rate determining the ratio strength to weight of the material used for their production. The increasing use of thin-walled steel elements as the main structural elements simultaneously necessitates the development of quick and effective methods of their strengthening. In traditional engineering practice, common strengthening methods are often associated with the need to modify the static scheme of the structure, increase the cross-section of structural elements, or increase the global stiffness of the structure. Among others, such an approach is described by the example of strengthening steel telecommunications towers, presented in [1]. However, not always such a wide scope of works is required or at all possible to strengthen steel structures. For example, a much simpler method is the application of CFRP (Carbon Fibre Reinforcement Polymers/Plastics) composite tapes to increase the load capacity of steel structures, which high effectiveness is confirmed by more and more frequent scientific publications. For example, the authors in [2] conducted an axial compression test of short round-section specimens reinforced with CFRP and without reinforcement. The authors in [3] presented a study of a thin-walled steel beam with a hat cross-section with a bonded steel plate in such a way as to obtain a closed cross-section. The beam was covered with a laminate made of CFRP fibres and then subjected to axial compression. The aim of the study was to analyse the cracking process in the CFRP layer and in the adhesive layer. Axially compressed thin-walled C-section reinforced with CFRP bars have been described in [4,5]. In [6], the behaviour of damaged steel thin-walled I-beams retrofitted with a carbon fibre mat (CFRP) is discussed. The possibility of optimising the design is also a common problem [7]. The work [8] presents a fatigue test of thin-walled butt-welded steel plates reinforced with CFRP sheets. In each of the works, the use of CFRP had a positive effect on the load-bearing capacity and stability behaviour of the analysed elements. In order to better understanding the specifics of the structural behaviour of composite materials, compression tests were also carried out on composite columns made of CFRP [9], and a series of studies on the CFRP-steel connection was described in [10].

Unfortunately, in the available literature, there is a shortage of works devoted to the steel thin-walled cold-formed beams subjected to bending reinforced by CFRP. Among the others, few include publications on research and numerical analyses of even unreinforced beams made of cold-formed Z [11,12,13], channel [14], or sigma [15] cross-sections. Recently, there have also been some works demonstrating bending in the case of composite beams [16].

The authors of this study have already published a pilot study of the methods of strengthening cold-formed sigma beams with CFRP tapes subjected to bending under a uniformly distributed load [17]. In this article, the presented study describes one of the stages of the research program concerning the reinforcement of sigma beams with CFRP tapes subjected to four-point bending. The preparation of the beams and the description of the measuring systems used in laboratory tests are discussed in [18].

## 2. Laboratory Tests

**Figure 1:**
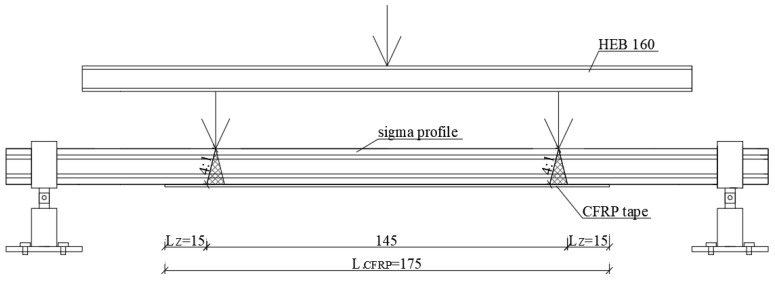
Scheme defining the determination of the carbon fibre reinforced polymer (CFRP) effective anchorage length.

The first step of this work was to determine the appropriate length of CFRP tapes. The issue is quite complex because there is no strict and universal recommendation describing the procedure of adoption of CFRP effective anchorage length. Therefore, based on the conducted thorough literature review in this study, the effective anchorage length was assumed in accordance with [19] and shown in Figure 1, where *L*_CFRP_ is the CFRP tape length, and *L*_z_ is the effective anchorage length of the CFRP tape. The length of the CFRP tape was of 175 cm and the effective anchorage length was of 15 cm. More information on the anchorage length tests is given in [20].

**Figure 2:**
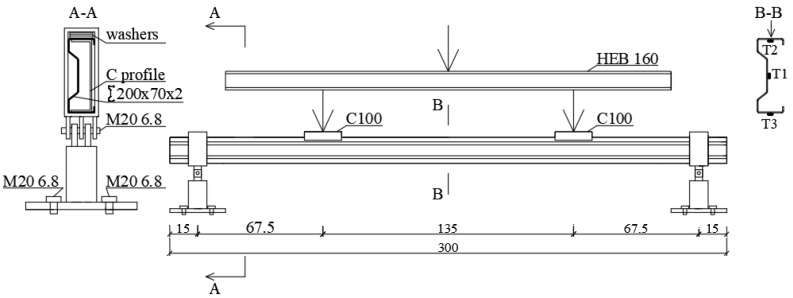
Laboratory stand scheme and location of measurement points.

Four-point bending laboratory stand was developed in order to perform full-scale tests. Experiments were carried out on a steel thin-walled, cold-formed ∑200 × 70 × 2 profile made by “Blachy Pruszyński” company. The load spacing of 135 cm and supports spacing of 270 cm was assumed (Figure 2). In order to determine the strength properties of the steel material, laboratory coupon tests were carried out on five samples cut out from sigma steel profiles. The shape and size of samples used for laboratory tests complied with the requirements of PN-EN ISO 6892-1: 2009 [21]. Measurements of the longitudinal and transverse deformation of the sample were carried out using a biaxial extensometer. Based on obtained results, material characteristic of ∑ profile such as the Young’s modulus *E* = 201.8 GPa, Poisson’s ratio *ν* = 0.28, and the yield strength of steel *f*_y_ = 418.5 MPa were specified. Sika CarboDur S carbon fibre tapes (CFRP), 1.2 mm thick and 50 mm wide, were used in the tests. Composite CFRP tapes used in the study consist of unidirectional arrangement of carbon fibres embedded in an epoxy matrix. One of the inherent features of this structure is anisotropy. Composite tape in different directions is characterized by different stiffness and strength. In the longitudinal direction, the stiffness and strength are very high, while transversely, the stiffness and strength are much weaker. The transverse modulus of a unidirectional laminate is only two to three times greater than that of the adhesive matrix itself, as is the strength, and in some cases even lower. On the basis of material tests of CFRP tapes, the Poisson’s ratio *ν* = 0.308 and Young’s modulus *E* = 165 GPa were determined. More information on the tape strength parameters is described in [22]. SikaDur^®^-30 adhesive was used in course to bond CFRP tapes to the beams. This adhesive was characterised by minimum compressive strength of 75 MPa after 7 days, a modulus of elasticity under compression of 9600 MPa, a minimum tensile strength after 7 days of 26 MPa, a deboning strength from steel after 7 days minimum 21 MPa, shear strength minimum 16 MPa, and shrinkage of 0.04%. Based on the tests presented in [19], the adhesive thickness was chosen to be equal to 1.3 mm. The author of this article [19], concerning the reinforcement of I-section steel beams with CFRP tape, examined three thicknesses of the adhesive layer—0.65 mm, 1.3 mm, and 1.75 mm. During the test, beams reinforced with CFRP tape with an applied adhesive layer of 1.3 mm achieved the highest value of the debonding force. Beams reinforced with CFRP tape with an adhesive layer of 1.75 mm achieved a lower value of the debonding force, which is due to the fact that in all samples the tape detached.

The first step of the samples’ preparation consists of degreasing and matting with sandpaper and cleaning the places where CFRP tapes were foreseen to be bonded. Detailed description of the preparation of samples for testing is provided in [18].

**Figure 3:**
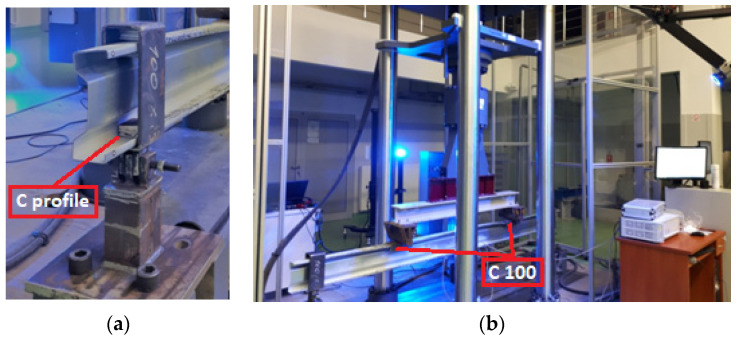
Four-point bending laboratory stand: (**a**) detail of beam support; (**b**) overall view of laboratory stand.

**Figure 4:**
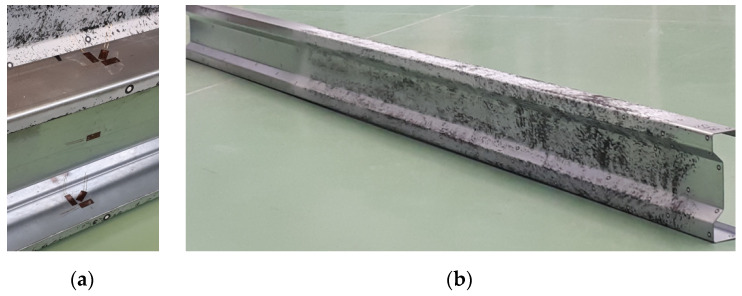
Samples preparation: (**a**) location of Tritop measuring points and electrofusion strain gauges; (**b**) view of samples with matt black and white pattern.

**Figure 5:**
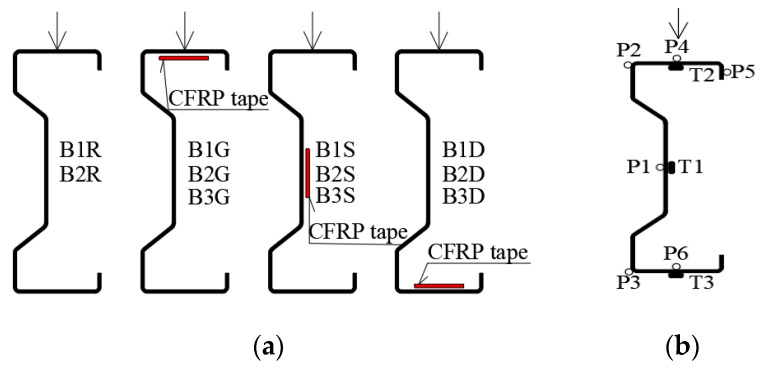
Scope of the tests: (**a**) steel tapes location and sample symbols; (**b**) measuring points in the middle span of the beam (electrofusion strain gauges—T1, T2, T3; displacement measurement—P1, P2, P3, P4, P5, P6).

So-called fork boundary conditions at laboratory circumstances was obtained by usage of a bolted hinge connection at the support, which enables free rotation in the beam plane. Additionally, special washers were applied to the beam at the points of concentrate forces occurrence. They were introduced in order to prevent local damage of the tested sigma beams. Such points were detected at the support region (Figure 3a), where the hot-rolled C profile was used, and at the point of application of external concentrated force, where the hot- rolled C100 profile with length of 200 mm was applied to ensure the load distribution over the entire flange area (Figure 3b). The process of sample preparation were shown in Figure 4. The Zwick and Roel (ZwickRoell GmbH & Co. KG, Ulm, Germany) testing machine at the Construction Laboratory of the Lublin University of Technology was used in order to perform experimental tests. The load increase was controlled by means of the extending piston press at a speed of 1 mm/min, recording the force every 0.01 s. During laboratory tests, strain measurement was carried out using three electrofusion strain gauges type TENMEX TFs-10 with 120 Ω ± 0.2% resistance. In each sample, the electrofusion strain gauges (T1, T2, T3) were located in the middle of its span. The arrangement of these gauges is presented in Figure 2—Section B-B and in Figure 5b. On the other hand the displacements were measured using the Aramis system and GOM Correlate software. For this purpose, a unique combination of two Aramis coupled devices and a Tritop machine was used, which enabled precise measurement of the displacement of the beam subject to significant rotation. The displacements of the specimens were measured at six measuring points (P1, P2, P3, P4, P5, P6) located in the middle of the beam span and affixed with special markings shown in Figure 5b.

The use of the Tritop (GOM, GmbH, Braunschweig, Germany) and Aramis (GOM, GmbH, Braunschweig, Germany) optical measuring systems requires that the steel surfaces of the tested beams do not reflect light. Consequently, all samples were painted with matt white spray paint. Then, the surface was additionally matted by spraying white chalk on it (Figure 4b).

Measuring points with a diameter of 5 mm were placed on the beam walls, (Figure 4a), which allowed for preliminary measurements of geometric imperfections [23] using the Tritop system. Moreover, they were used to create a common coordinate system for the two measuring lenses in the Aramis system [24]. These points were also used to analyse the displacements of the tested beams in the GOM Correlate program (GOM, GmbH, Braunschweig, Germany). As it was mentioned before, the conducted research used optical 3D coordinate measuring machine—Tritop. It is a portable system enabling precise and quick measurements of 3D coordinates of objects. A series of accurate photos of each of the tested beams was taken using a camera. The photos were transferred to the GOM Correlate program and measurements of individual beam dimensions were made. Section height, top flange width, and bottom flange width were monitored. All measured values were within dimensional tolerances (1 mm for height and 0.5 mm for width). Therefore, in the numerical study the impact of imperfection was neglected. Rules of application and use of the Aramis and Tritop system are in details described in [18].

The scope of this part of the laboratory research included eleven steel beams made of ∑200 × 70 × 2 profile with the span of 270 cm. In order to investigate the influence of CFRP tape location, three beams ware reinforced with CFRP tape placed on the inner surface of the upper flange (B1G, B2G, B3G), three with CFRP tape bonded on the inside surface of the web (B1S, B2S, B3S), three beams with reinforcement located on the inside surface of the bottom flange (B1D, B2D, B3D). To obtain information on the effectiveness of the applied reinforcement, two unreinforced beams (B1R, B2R) were taken as a reference beams (Figure 5a).

Based on the laboratory tests it was observed that failure mode of all beams was related to debonding of CFRP tapes in the adhesive-steel contact plane in the load range within 25–26 kN (Table 1). Moreover, it was noted that the correct reading of all electrofusion strain gauges was possible up to load of 25 kN, due to debonding of the CFRP tape. Therefore, it was assumed that this load level should be considered as the destructive force. As a consequence, in the further analysis, this load level was adopted as the limit load, and the final CFRP tapes performance reducing strain and displacement of tested beams was described at this load level.

**Figure 6:**
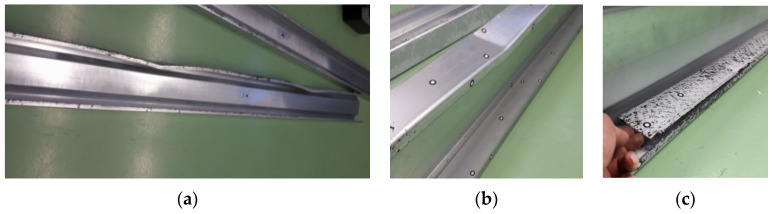
Form of sample failure mode: (**a**) local damage of top flange; (**b**) mixed failure mode: local damage of top flange and debonding of the CFRP tape; (**c**) debonding of the CFRP tape.

Simultaneously, debonding failure mode and the deformation in form of opening of the beam cross-section was observed. Namely, the top flange lifted between the load application points and caused the reasonably large displacement out of the vertical beam plane. Moreover, local upper flange deformations were observed at the points of concentrate load occurrence. At the same time, the bottom flange was not damaged. The nature of beam deformation and the debonding of the CFRP tapes are shown in Figure 6.

**Figure 7:**
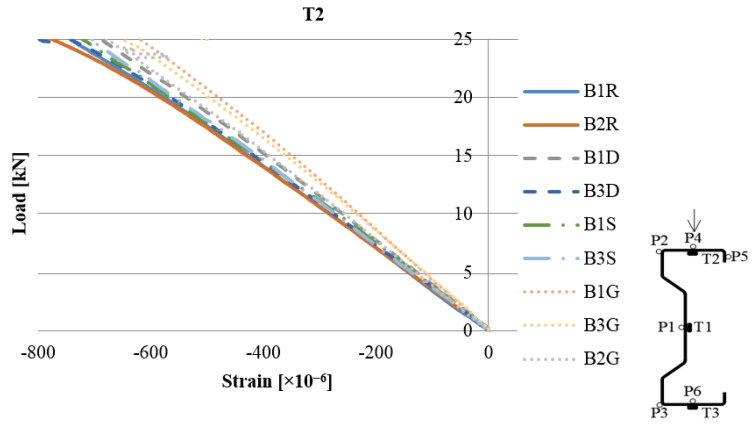
Load–strain diagram based on the reading from the T2 strain gauge.

**Figure 8:**
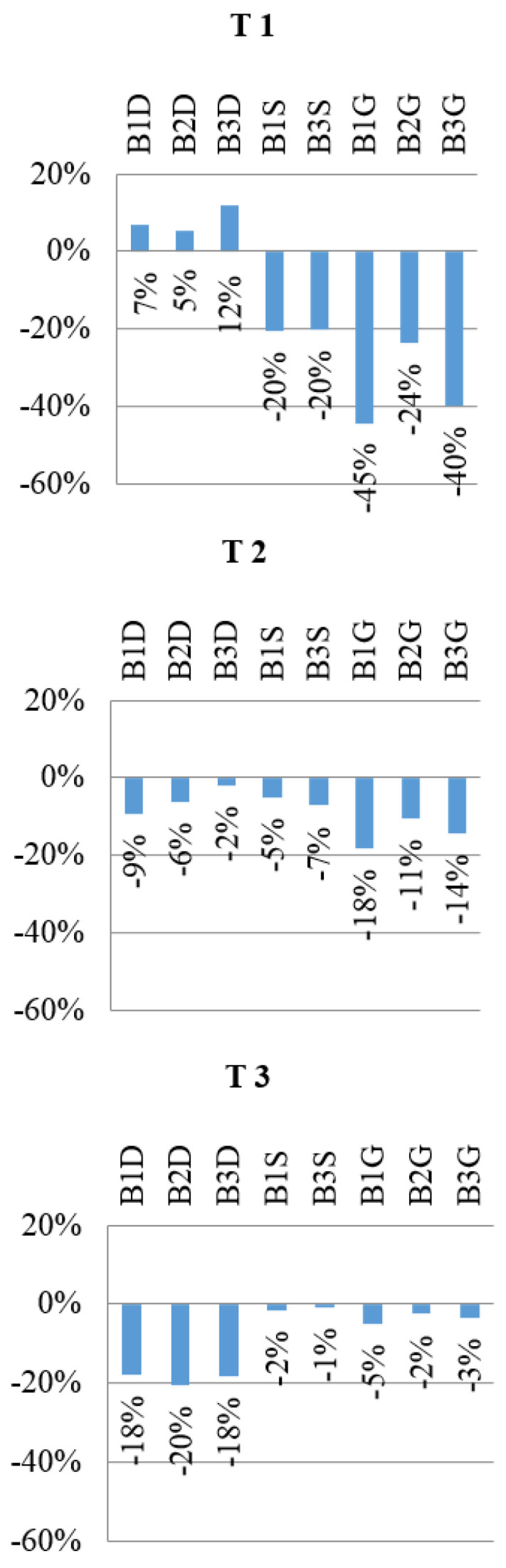
Readout from strain gauge at 25 kN load level. Percentage change in strain for different CFRP tape location in reference to bare beams (without reinforcement).

The exemplary load–strain relationship obtained during the laboratory test is shown in Figure 7. To enable the analysis of the obtained results, bar graphs were prepared, which are presented in Figure 8. The given values were determined on the basis of the following Equation (1):

(1) $$\rho_{\varepsilon i}\;=\;\left(\frac{\varepsilon_{i}}{\varepsilon_{\mathrm{ref}}}-1\right)\times 100\%$$

where:${\;\rho }_{\varepsilon i}$—reduction or increase in strain of a given sample expressed as a percentage, ${\;\varepsilon }_{i}$—strain of a given sample, $\varepsilon_{\mathrm{ref}}$—arithmetic mean value of the strain of two reference beams (B1R and B2R).

**Figure 9:**
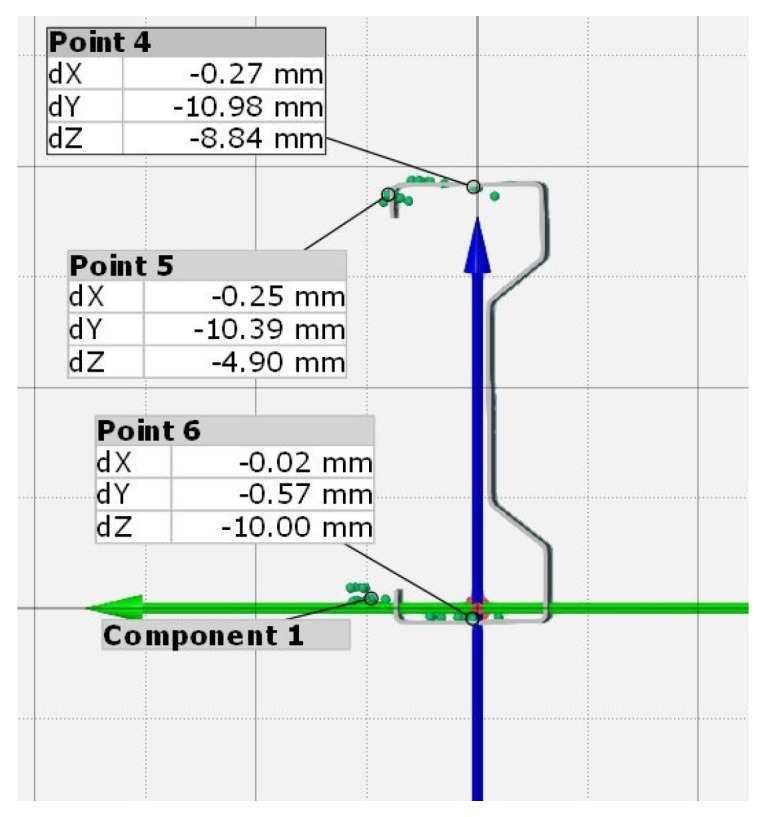
Displacements of selected cross-section points in the middle of the beam span under a load of 25 kN.

The displacement of selected points located in the mid-span of example sample under the load of 25 kN, obtained with the GOM Correlate software, is shown in Figure 9.

Using Equation (2), the percentage change in displacement taking into account different location of reinforcement with CFRP tape versus reference bare beams at the load level of 25 kN was determined.

(2) $$\rho_{\mathrm{ui}}\;=\;\left(\frac{u_{i}}{u_{\mathrm{ref}}}-1\right)\times 100\%$$

where:${\;\rho }_{\mathrm{ui}}$—percent change in displacement of the ith sample,${\;u}_{i}$—displacement of the ith sample, $u_{\mathrm{ref}}$—displacement of reference beam.

**Figure 10:**
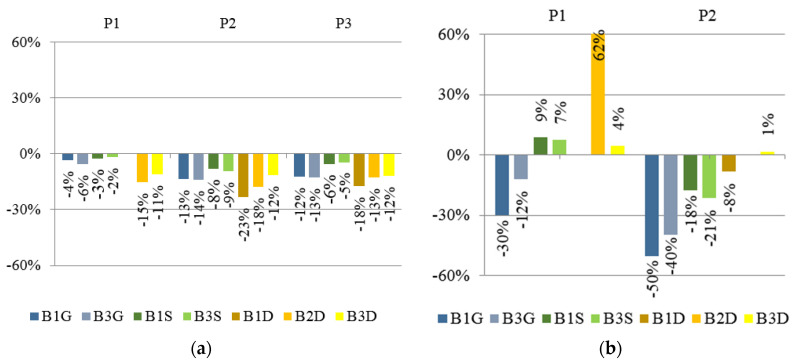
Percentage change for different location of CFRP tape in reference to bare beams (without reinforcement) in: (**a**) vertical displacement; (**b**) horizontal displacement. Readout from points P1, P2, and P3 at 25 kN load level.

The percent change in vertical displacement is presented in Figure 10a and for horizontal displacement in Figure 10b. Positive values described increase in displacement, while negative values indicate a reduction in displacement.

## 3. Numerical Study

**Figure 11:**
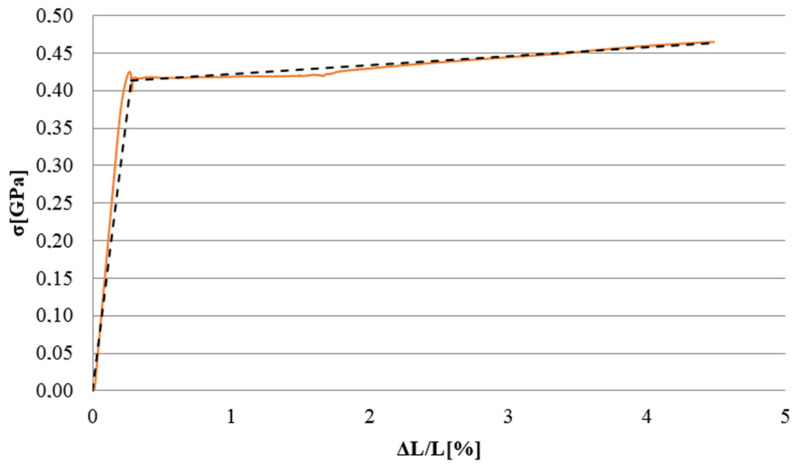
Material characteristics of the specimen made of steel: laboratory coupon test and numerical model.

The numerical model was developed in Abaqus (Abaqus 2019, Dassault Systemes Simulia Corporation, Velizy Villacoublay, France). The experimentally analysed sigma shaped beam was described with a discrete model, where the tested profile was subjected to numerical analysis of the beams subjected to bending, due to the applied loads [7,25]. The finite element model of the sigma steel beam, respectively, for the laboratory tests, is made of shell finite elements (linear shape function). With reference to the numerical model, both the steel washers and steel support clamps, cooperating with the steel beam reinforced with composite tapes, were made of non-deformable shell finite elements. Inside the beam profile, at the opposite ends of the profile, there were channel profiles made of steel to reduce local deformation of the beam profile. The C-profile was made as a shell finite element with a defined thickness. The material model within the discrete model was prepared on the basis of information from experimental tests [9]. On the basis of the σ-ε relationship obtained in laboratory coupon test, the bilinear elastic–plastic material model with strain hardening was adopted in the FEM numerical model. The material properties were as follows: Young’s modulus (201.8 GPa), Poisson’s ratio (0.282), and yield strength (418.5 MPa) (Figure 11). The lower value of Young’s modulus than for the S350 GD steel grade results from the fact that the samples are made of galvanised steel.

**Figure 12:**
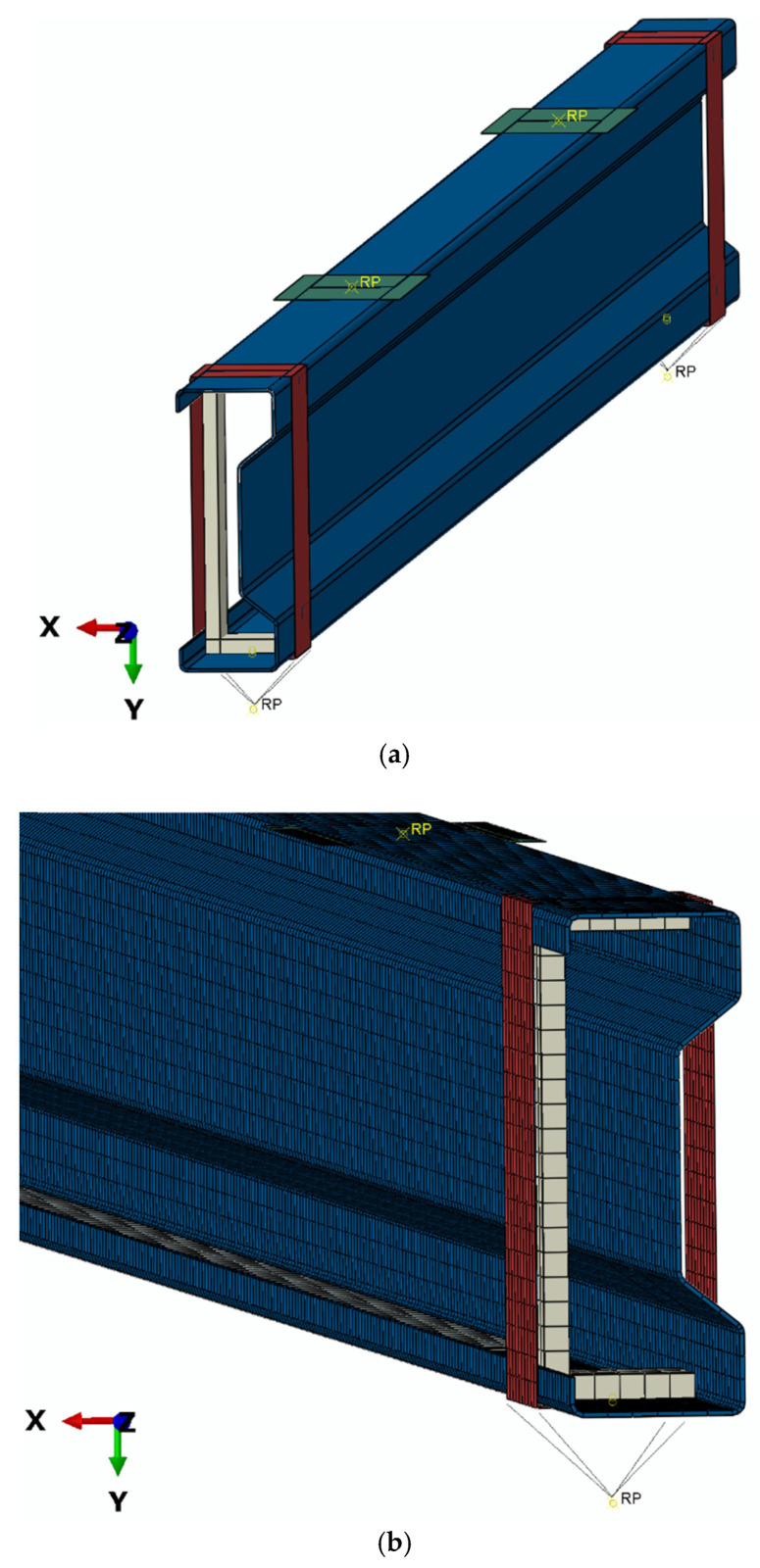

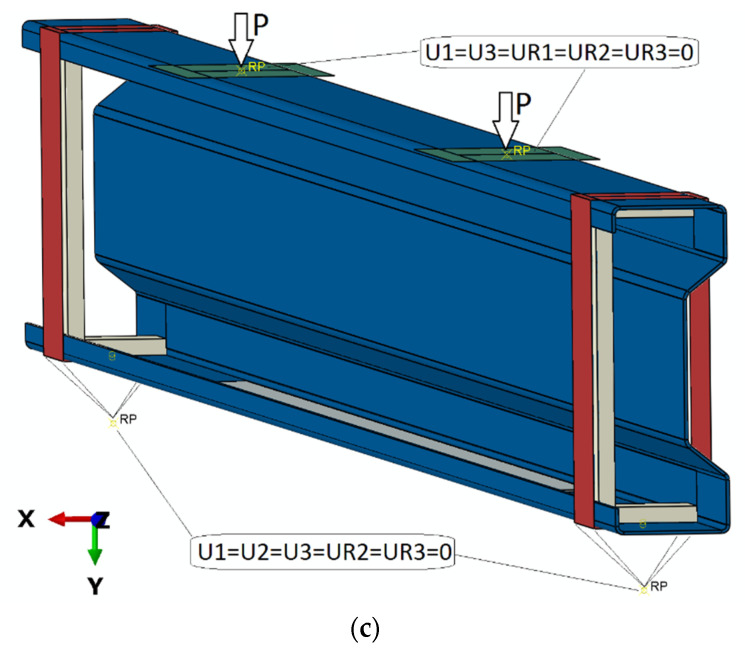
Numerical model of beam: (**a**) shell model; (**b**) discrete model; (**c**) boundary conditions of model.

The research has mapped as accurately as possible the boundary conditions resulting from the experimental studies. For both non-deformable steel support clamps and steel washers, appropriate reference points have been defined in order to allow further declaration of the necessary boundary conditions. In these reference points assigned to the steel washers, a load of equal value is defined for each of the non-deformable steel washers (as shown in Figure 12). For the non-deformable steel support clamps, the necessary boundary conditions are also defined at the reference points (as shown in Figure 12). The numerical model was prepared taking into account contact interactions in normal and tangential directions, without taking into account the friction coefficient. In order to achieve the appropriate mapping of the experimental studies, contact relations were defined between the beam and the support clamps, channel sections, and washers (Figure 12). The finite element model included 18,312 computational nodes, with a number of finite elements equal to 17,713. The number of deformable shell elements was equal 16,273 (for beam and a channel bar). The number of non-deformable linear shell elements was equal 1440 (for supports). In numerical tests, a bi-linear elastic–plastic material model was used for beam specimen. CFRP tapes were modelled as shell finite elements connected to the beam by TIE joints. The material model used to describe CFRP tapes had orthotropic properties (*E*_1_ = 142 GPa, *E*_2_ = 8 GPa, *ν*_12_ = 0.308, and *G*_12_ = *G*_23_ = *G*_13_ = 4.5 GPa). The preliminary research focused on the preparation of a bare-beam model, in order to validate the model by performing tests on actual samples. Further research objectives included the development of appropriate three subsequent numerical models, after obtaining agreement of results for this basic case. Namely, model: BGa with CFRP tape placed in the upper flange (analogously to the B1G, B2G, B3G tested in the experimental study), BDa with CFRP tape in the bottom flange (like B1D, B2D, B3D), and the BSa with CFRP tape in the web (corresponding to B1S, B2S, B3S).

**Figure 13:**
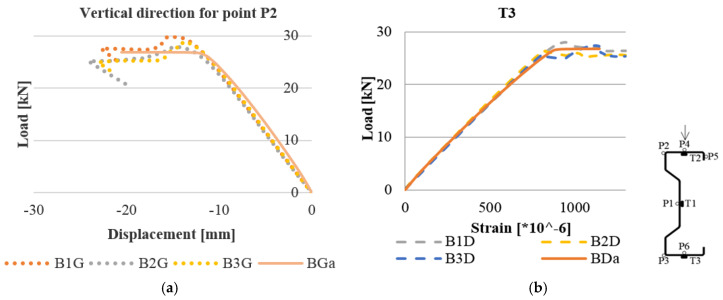
Comparison of laboratory and numerical tests at an analogous point for reinforced beams in the web: (**a**) load–displacement for point P2 in the vertical direction; (**b**) load–strain for the T3 strain gauge.

A detailed analysis of the obtained test results was carried out mainly in places where electrofusion strain gauges T2 and T3 were initially placed, regarding the laboratory tests (the locations of strain gauges are shown in Figure 13b). The strain level that was read in the laboratory tests was directly compared with the results of numerical calculations, precisely with Max. In-Plane Principal (Abs) from Abaqus. These are components illustrating strains in the longitudinal direction of the beam in the plane of the individual walls of the section. In the numerical analysis, vertical displacements and horizontal displacements of the beams were reported at the points where measurements were made during laboratory tests. Examples of strain and deformation comparison of selected beams are presented in Figure 13a,b.

**Figure 14:**
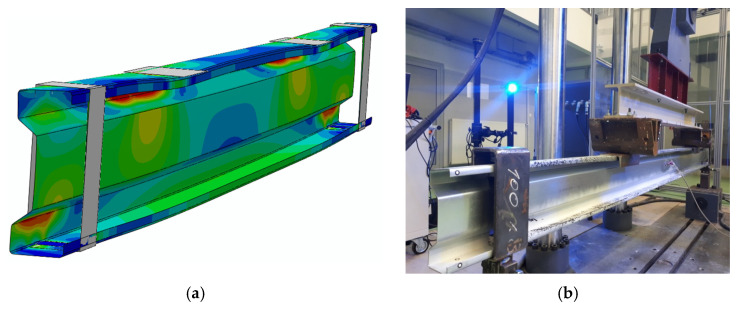
Beam deformation: (**a**) numerical analysis; (**b**) laboratory tests.

In the case of strains, the maximum discrepancies between the results obtained in the Abaqus program and the average value of measurements for a given group of beams in a laboratory test reach a maximum of 4.1% at a load level of 25 kN, and in the case of displacements, 4.6–6.8%. The form of beam deformation observed during laboratory tests is also consistent with the shape of deformation obtained in the Abaqus program (Figure 14). On the basis of further research stages, it was found that the described model is also applicable for various sigma cross-section heights.

## 4. Results and Discussion

Regarding to the obtained results, a quite good agreement between FEM analysis and experimental tests were obtained. In the presented work, the retrofitting method of thin-walled, cold-formed sigma beams using bonded CFRP tapes is proposed. The study investigated the effectiveness of the applied reinforcement, where satisfactory results were obtained.

As a consequence of bonding the CFRP tape to the lower or upper flange of the beam an increase in the moment of inertia with respect to the y axis by 18%, and with respect to the z axis, by nearly 9%, and the change in position of the gravity centre of the cross-section is observed by 1.26 mm in the horizontal direction and by 13.6 mm in the vertical direction. It is worth to note that placing the tape in the lower flange allows us to reduce the vertical displacement of the beam by 23% and the horizontal displacement by 8%, and the use of CFRP tape in the upper flange allows us to reduce the vertical displacement of the beam by 14% and horizontal displacement by up to 50%. One can notice that the increase in the percentage of reinforcement of the analysed beams is not only the result of an increase in the geometric characteristics of the beam and CFRP tape system, but also it is a beneficial influence of the proposed method.

It is not surprising that the use of a reinforcement in the web of the beam limits vertical displacements to the least extent. It is worth emphasizing, however, that the use of web reinforcement, which changes the moment of inertia by no more than 0.5%, reduces vertical displacements by 9% and horizontal displacements by up to 21%.

It should be also mentioned that the proposed reinforcing method is characterized a significant deviation from the classic methods of strengthening steel building structures. Namely, because of technological limitations resulting from the access to the reinforced element “in situ”, proposed method consciously departed from the principle of the coincidence of the centres of gravity of the basic section and the reinforcement sections. It results from the specific geometry of the sigma section itself, which has only one axis of symmetry.

As noted in [19], the strength of steel is higher than that of conventional adhesives used in structural strengthening applications, which results in a variety of possible forms of failure. Cohesive failure in the adhesive layer, adhesive detachment along the surface of the adhesive–composite or steel–adhesive interface, Fiber Reinforced Polymer (FRP) delamination are possible forms to be considered when designing a steel reinforcement with such a structure.

In this study, all the steel beams were reinforced with one thickness of the adhesive layer (1.3 mm), and in each of the samples the tape was debonded at the glue–steel interface. Therefore, the authors cannot conclude that the use of a different thickness of the adhesive layers will change the debonding effect. It is suspected that debonding was due to the significant local deformation of the beams and not to the thickness of the adhesive. Due to the many possible forms of failure and incomplete knowledge of the behaviour of composite materials adhered to steel, it will be desirable to carry out more research before widely adoption of proposed method into engineering practice.

## 5. Conclusions

Based on the obtained laboratory and numerical results the beneficial influence of CFRP tapes on the displacement and strain reduction in case of thin-walled cold-formed beams made of 200 × 70 × 2 is observed.

This is confirmed by the detailed conclusions that flow from the individual analyses, and which can be expressed as follows:

- The average percentage reduction in strain in upper flange (14%) and in web (36%) is achieved for CFRP tape located in the upper (compressed) flange.
- The 18−20% strain reduction in the bottom flange is observed when CFRP tape is bonded to bottom flange.
- The decrease in vertical displacements in average value of 11–23% is obtained when the CFRP tape is placed on the bottom flange.
- The decrease in horizontal displacement perpendicular to the longitudinal axis of the beam in the upper flange by 50% for reinforcement CFRP tape located on the upper (compressed) flange is achieved.
- Location CFRP tape on the bottom flange did not reduce the horizontal displacement in any case.
- Web reinforcement results in reduction in horizontal displacement in the upper flange by 18–21%, and of vertical displacement by 9%.

Summing up, it was found that the location of CFRP tape at the upper flange and at the web can be very advantages in case of the beam subjected to the large torsion. The innovative solution proposed in this paper is the placement of the CFRP tape on the inside surface of the flange, which is easier from technological point of view during construction works. In addition, authors used the innovative methods of displacements measurement using two lenses of the Aramis system, positioned on both sides of the tested beam, in combination with the Tritop system, which enabled the study of the displacement of mono-symmetrical beams in 3D.

Finally, it can be stated that the traditional, well-known engineering strengthening method, which recommend bonding the CFRP tapes to the bottom (tensioned flange), as the beams under consideration cannot be considered as a universally favourable.

## Figures and Tables

**Table 1:** Form of sample failure mode.

No. of Sample	Failure Mode	Debonding Load Level [kN]
B1D	Full debonding of CFRP tape	25.6
B2D	One side debonding of CFRP tape on a considerable length (about 40%)	26.1
B3D	One side debonding of CFRP tape on a considerable length (about 40%)	26.3
B1S	Both side debonding on about 30% of length of CFRP tape	26.8
B2S	Lack of CFRP tape debonding	-
B3S	Slight one side debonding of CFRP tape (about 10%)	26.2
B1G	Slight one side debonding of CFRP tape (about 10%)	25.4
B2G	Slight one side debonding of CFRP tape (about 10%)	26.0
B3G	Slight one side debonding of CFRP tape (about 10%)	27.1
